# Recent advances in nontuberculous mycobacterial lung infections

**DOI:** 10.12688/f1000research.20096.1

**Published:** 2019-10-01

**Authors:** David Horne, Shawn Skerrett

**Affiliations:** 1 Division of Pulmonary, Critical Care, and Sleep Medicine, University of Washington, Harborview Medical Center, Seattle, USA

**Keywords:** Non-tuberculous mycobacteria

## Abstract

Nontuberculous mycobacteria (NTM) are members of the Mycobacterium genus other than *Mycobacterium tuberculosis* complex and *Mycobacterium leprae*. NTM are widely distributed in the environment and are increasingly recognized as causes of chronic lung disease that can be challenging to treat. In this brief review, we consider recent developments in the ecology, epidemiology, natural history, and treatment of NTM lung disease with a focus on *Mycobacterium avium* complex (MAC) and *Mycobacterium abscessus* complex *.*

## Introduction

Nontuberculous mycobacteria (NTM) are members of the Mycobacterium genus other than *Mycobacterium tuberculosis* complex and *Mycobacterium leprae*. NTM are widely dispersed in natural and man-made environments, mainly in association with soil, water, and biofilms. To date, more than 190 species have been identified, most of which have not been linked with human disease. In this brief review, we consider recent developments in the epidemiology and treatment of NTM lung disease, the most common clinical presentation of NTM infection. We focus our discussion on *Mycobacterium avium* complex (MAC) and *Mycobacterium abscessus* complex. *M. avium* complex includes *M. avium* subspecies *Mycobacterium intracellulare* and *Mycobacterium* *chimaera*. *M. abscessus* complex comprises three distinct subspecies: *M. abscessus* subspecies *abscessus*, *M. abscessus* subspecies *bolletii*, and *M. abscessus* subspecies *massiliense*.

## Ecology and epidemiology

### Varied geographic distribution

A key feature of NTM is variability in their geographic distribution. A global survey of NTM species isolated from human specimens found that almost one-half were MAC, although the relative frequency varies widely by geographical region (e.g. MAC represented 31% of isolates from South America, 52% from North America, and 71% from Australia) ^1^. A study of US clinical isolates from 2009 to 2013 also demonstrated regional variability in the distribution of NTM species ^2^. MAC accounted for 61–91% of total NTM isolates and were most frequent in the South and Northeast of the US, while the rapid growers *M. abscessus* and *Mycobacterium chelonae* represented 2–18% of isolates and were most frequent in the West. A study of NTM isolates in Washington State found that unusual NTM species in the US were more commonly recovered from non-US-born individuals and reflected the distribution of NTM in their countries of origin ^3^. This finding may be consistent with a latency phenomenon in NTMs similar to *M. tuberculosis* ^4^.

### Increasing prevalence

Studies from multiple countries indicate that the incidence of NTM infection is increasing globally ^5, 6^ and that MAC infections are the main driver of this increase ^7^. Heightened clinician awareness, more sensitive chest imaging, and improved culture techniques all may contribute to increased recognition of NTM infection, but the evidence supports a true increase in the burden of NTM disease. Data from the National Health and Nutrition Examination Survey (NHANES) demonstrated an increase in cutaneous delayed-type hypersensitivity to *M. intracellulare* in the US from 1970 to 2000 ^8^. In Japan, NTM-related deaths increased from 1970 to 2010 ^9^. A number of factors may underlie the increased prevalence of NTM infections. An aging population with relevant co-morbidities may be more vulnerable to NTM disease ^10^. In addition, contemporary water treatment strategies can select for NTMs in potable water systems, potentially increasing environmental exposure.

The prevalence of pulmonary NTM infections appears to be increasing among patients with cystic fibrosis (CF). Data from the Cystic Fibrosis Foundation Patient Registry revealed that the annual prevalence of positive sputum cultures for NTM increased from 11% in 2010 to 13.4% in 2014 ^11^. Among patients with NTM, MAC was isolated from at least one specimen in 61%, *M. abscessus* in 39%, and other NTM in 21%; 19% of patients had multiple species isolated ^11^. The clinical significance of these isolates is not clear ^12^. In one recent study of 96 CF patients with at least one positive sputum culture for NTM, only 37 cases met ATS criteria for active NTM lung disease ^13^. However, an accelerated decline in lung function was observed among those with NTM lung disease ^13^. Furthermore, isolation of *M. abscessus* from CF patients is commonly considered a contraindication to transplantation because of poor outcomes ^14^.

### Person to person transmission?

Careful analysis of *M. abscessus* isolates from CF patients has suggested the possibility of direct or indirect person-to-person transmission of NTM, counter to the prevailing paradigm that NTM infections are acquired only from environmental sources. Following the report of an outbreak of *M. abscessus* subspecies *massiliense* infections among five patients in a CF center ^15^, a number of studies have used whole-genome sequencing and phylogenetic analysis to investigate the genetic epidemiology of *M. abscessus* ^16^. A recent global study of 1,080 clinical isolates of *M. abscessus* harvested from 517 patients at multiple CF centers on three continents found that the majority of infections worldwide were caused by genetically clustered organisms, a pattern that suggested recent transmission rather than independent acquisition of unrelated strains ^17^. A panel of these clustered isolates was found to exhibit increased intracellular survival in macrophages and enhanced virulence in a murine model of infection when compared to unclustered isolates ^17^. These data suggested the possibility that dominant clones of *M. abscessus* might be transmitted by fomites or persistent infectious aerosols. However, this model remains controversial ^16, 18^.

### Increased mortality

A systematic review of mortality in patients with MAC lung disease found that all-cause 5-year mortality varied from 10–48% and that MAC-related mortality was 5–42% ^19^. There was high heterogeneity across studies, and the effect of medical treatment was not estimated. Given the presence of older age and co-morbidities in many patients with NTM, comparing mortality rates to matched controls is important for developing accurate estimates of the impacts of NTM disease on survival. A population-based study from Ontario, Canada, identified more than 18,000 patients with NTM pulmonary isolation, categorized patients as having lung disease (more than one positive sputum sample) or infection (one positive sputum sample), and matched them to residents of the province without NTM using propensity scores ^20^. The standardized mortality ratios (SMRs) were 2.59 (95% confidence interval [CI] 2.49–2.69) and 2.27 (2.16–2.38) for participants with disease and infection, respectively. For *M. abscessus* lung disease, the SMRs were 2.23 (95% CI 1.71–2.74) and 2.10 (1.46–2.74). As this study was based on administrative data, the authors were unable to identify the cause of death and determine whether the relationship between sputum isolation of NTM and decreased survival is associative or causal. A similar cohort analysis of a US insurance database compared 2,005 individuals with NTM lung disease to age-matched controls and found that adjusted all-cause mortality was more than twofold higher in the NTM population ^21^.

Studies suggest that mortality rates are fivefold to eightfold higher in individuals with cavitary MAC lung disease compared to nodular-bronchiectatic lung disease ^19, 22– 24^, and this association persisted in multivariable models that adjusted for co-morbidities such as emphysema ^23– 25^. The natural history of nodular-bronchiectatic MAC lung disease is less certain, with studies suggesting that almost one-quarter of patients will have radiologic deterioration ^26^ and almost one-half require treatment initiation ^27^ over 2–5 years of follow-up. A recent retrospective study assessed patients with nodular-bronchiectatic MAC lung disease who were not treated with antibiotics for at least 6 months after diagnosis ^28^. Over a mean follow-up period of 6.9 years, 23% of participants were started on treatment. Among the untreated participants, mean body mass index (BMI) and the ratio of forced expiratory volume in 1 second to forced vital capacity (%FEV _1_, a measure of obstructive lung disease) declined, and bronchiectasis worsened significantly. In the treatment group, bronchiectasis also significantly worsened, although BMI and %FEV _1_ remained stable. For patients with nodular-bronchiectatic lung disease who are not ready to start treatment, assessing changes in spirometry values over time may help identify those who would benefit from antibiotic therapy.

## Treatment

### Standardized definitions of treatment outcomes

The decision to initiate antibiotic treatment for MAC lung disease is based on patient symptoms, radiographic findings, evidence of disease progression, and patient preferences ^29^. In 2018, a consensus study by US and European pulmonary and infectious diseases societies on outcome definitions for NTM lung disease was published ^30^. Although designed to improve research including comparisons between studies, the statement includes points that are relevant to clinicians. Culture conversion was defined as the finding of at least three consecutive negative mycobacterial cultures collected at least 4 weeks apart (the sampling date of the first negative culture is then the date of culture conversion) ^30^, which was not fully defined in the ATS guidelines ^29^. For patients who have experienced culture conversion, a single (isolated) positive culture after culture conversion does not demonstrate treatment failure and may be due to re-infection with a new strain ^31^. The statement defined treatment failure as the re-emergence of multiple positive cultures or persistently positive cultures after 12 months or more of treatment. There is controversy around this timing (e.g. versus using a 6-month cut-off) and the statement notes that microbiological response after 6 months of treatment is a very accurate predictor of treatment failure (non-response) at 12 months ^32^.

### Guideline-based treatment outcomes

Guideline-based treatment (GBT) for nodular-bronchiectatic MAC lung disease includes a newer generation macrolide, ethambutol, and rifamycin ^29^. A recent systematic review of treatment outcomes in patients with MAC lung disease who were treated with a macrolide-based regimen identified 42 studies that met inclusion criteria, the majority of which were from the US (n = 15) and Japan (n = 15) ^33^. Many of the studies pre-dated the ATS/IDSA guidelines on NTM treatment, explaining, in part, that only 15 of the 42 studies (36%) used GBT. Among the studies (n = 7) that enrolled treatment-naïve participants with macrolide-susceptible isolates and used GBT for >12 months, the pooled treatment success rate (defined as sputum culture conversion that persisted throughout follow-up) was 66% (95% CI 53–77%). In comparison, the treatment success rate across all 42 studies was 53% (95% CI 46–60%). A separate systematic review that evaluated MAC lung disease outcomes when patients received macrolide-containing regimens found lower success rates in patients with cavitary disease (57%) compared to nodular-bronchiectatic disease (66%) and among patients with a previous treatment history (58%) compared to treatment-naïve patients (64%) ^34^. Differing from these meta-analyses, several large cohort studies showed high rates of successful treatment outcomes in patients with nodular-bronchiectatic MAC lung disease who received more than 12 months of the recommended three-drug therapy (>88%) ^35– 37^. Although treatment outcomes in MAC lung disease are less than ideal, adherence to GBT offers the best opportunity for cure.

A study of patients with refractory MAC pulmonary disease who had persistently positive sputum cultures after 12 months or more of GBT found that this is frequently due to re-infection with a new strain ^31^. Only 27% of patients had persistent infection with solely the original MAC strain, while 49% were culture positive with a new strain and 24% had mixed infection with both an original and a new strain. Re-infection occurred in both patients with nodular-bronchiectatic and cavitary disease. Macrolide resistance developed in 22% of patients but was not the primary cause of refractory disease. These important findings highlight the difficulty in curing patients with MAC lung disease who are constantly at risk for re-infection.

### Evidence for guideline-based treatment

For patients with nodular-bronchiectatic MAC lung disease, ATS/IDSA guidelines recommend the use of a thrice-weekly regimen composed of a macrolide, rifampin, and ethambutol to balance treatment efficacy with medication tolerability ^29^. Given less than optimal rates of good treatment outcomes, many experts advocate for daily therapy in patients who do not achieve culture conversion. A small study evaluated the outcomes of patients who were switched to daily therapy after 12 months of GBT without culture conversion ^38^. They found that among 20 patients, six (30%) became culture negative after a median of 56 days on daily treatment. Among the patients without culture conversion, three out of four who underwent surgery also achieved culture conversion. Of note, the 30% culture conversion rate was not statistically different from patients who were continued on thrice-weekly therapy despite 12 months of culture positivity. A randomized controlled trial is currently comparing daily to intermittent treatment for nodular-bronchiectatic MAC lung disease ^39^.

The 2007 guidelines noted the lack of studies evaluating two- versus three-drug regimens for the treatment of MAC lung disease. An older study of HIV-positive individuals with disseminated MAC found that the two-drug regimen was associated with the development of macrolide resistance ^40^. However, studies of MAC lung disease suggested that ethambutol is the key co-drug in preventing macrolide resistance ^41, 42^. Japanese investigators randomized 119 participants to either a two-drug (clarithromycin and ethambutol, n = 60) or a three-drug (clarithromycin, ethambutol, and rifampin, n = 59) regimen ^43^. Sputum culture conversion rate in the three-drug regimen was 41% and in the two-drug regimen was 55%. The former exhibited adverse events resulting in treatment cessation at an incidence of 37% and the latter at an incidence of 27%. Eight patients (14%) in the three-drug regimen and seven patients in the two-drug (12%) regimen who did not reach sputum conversion gave isolates that still demonstrated clarithromycin susceptibility upon completion of the study. There is an ongoing multicenter study to evaluate whether a two-drug regimen for nodular-bronchiectatic MAC lung disease can increase tolerability without a substantial loss of efficacy (NCT03672630).

### Adherence to guideline-based treatment

The 2007 ATS guidelines included recommendations for the treatment of patients with NTM based on species and imaging findings ^29^. It is clear that the inclusion of a macrolide in MAC and *M. abscessus* subspecies *massiliense* treatment regimens greatly improves the likelihood of achieving cure. A nationwide survey of US physicians who treat patients with MAC and/or *M. abscessus* lung disease was conducted in 2011–2012 ^44^. Among patients treated for MAC, only 13% received an antibiotic regimen that met ATS/IDSA guidelines, 30% received a regimen associated with increased risk for macrolide resistance, and 56% received a regimen that did not include a macrolide. Among patients with *M. abscessus*, 64% of regimens prescribed did not include a macrolide. Similar findings were reported in a survey of clinicians from Europe and Japan, where 9% and 42% of patients treated for MAC lung disease received at least 6 months of a regimen containing a macrolide, ethambutol, and rifamycin, respectively ^45^. **

### Clinical phenotypes and outcomes

MAC species include *M. avium*, *M. intracellulare*, and *M. chimaera*, among other less clinically relevant strains. Similar to variations in the frequencies of NTM species, the frequency of MAC species varies geographically. For example, *M. avium* predominates in the Americas (64–78% of MAC isolates) and represents 47% of MAC isolates in Europe, while *M. intracellulare* is most frequent in Australia (80% of MAC) and South Africa (78% of the MAC) ^1^. This subspecies diversity is of more than academic interest, as growing data suggest that clinical and treatment outcomes vary by subspecies. Studies suggest that compared to *M. avium*, patients with lung disease due to *M. intracellulare* present with more advanced disease and are at greater risk for disease progression ^46, 47^ but are at lower risk for relapse or reinfection after cure ^46^; these findings may differ in areas where other MAC genotypes predominate ^48^. *M. abscessus* complex is differentiated into three species: *M. abscessus*, *M. massiliense*, and *M. bolletii.* Unlike the other two subspecies, *massiliense* lacks a functional *erm41* gene and remains susceptible to macrolide antibiotics ^49^. For this reason, cure rates in patients with disease due to *massiliense* (57%) are higher than rates in patients with *M*. *abscessus* (33%) ^50– 53^.

## Novel treatments

### Liposomal amikacin

Amikacin encapsulated in liposomes for inhalational administration (amikacin liposome inhalation suspension, “ALIS”) penetrates mycobacterial biofilms and increases amikacin uptake by alveolar macrophages compared to non-liposomal amikacin preparations. A phase III industry-funded international study investigated ALIS efficacy when added to GBT compared to GBT alone in those with refractory MAC lung disease, i.e. MAC positive while on GBT for at least 6 months ^54^. Patients with CF, immunodeficiency syndromes, or amikacin-resistant isolates (MIC >64 µg/ml) were excluded. The primary endpoint was culture conversion by 6 months of treatment. Significantly more participants who received ALIS achieved the primary endpoint (29%) compared to GBT alone (9%; adjusted odds ratio [OR] 4.2, 95% CI 2.1–8.6). The most common adverse events were respiratory related and occurred more frequently in the ALIS arm (87%) compared to GBT alone (50%), and 17% of ALIS-treated patients had adverse events that resulted in discontinuation of ALIS. ALIS is the first FDA-approved medication for the treatment of refractory MAC lung disease.

### Clofazimine

Clofazimine is an r-aminophenazone dye long used to treat leprosy. Clofazimine is also effective against *M. tuberculosis* ^55^ and is recommended by the World Health Organization for the treatment of multidrug-resistant tuberculosis ^56^. Clofazimine exhibits good activity against MAC, which may be more effective versus *M. intracellulare* than *M. avium* ^57^. A number of studies have evaluated clofazimine for the treatment of patients with NTM lung disease, both MAC ^58– 61^ and *M. abscessus* ^60, 62^, including refractory disease. A retrospective review showed higher culture conversion rates in patients with MAC lung disease treated with the addition of clofazimine to a macrolide and ethambutol (100%) compared to those treated with rifampin in addition to a macrolide and ethambutol (71%, *P* = 0.0002) ^59^. In the treatment of *M. abscessus*, 24% of patients achieved culture conversion after the addition of clofazimine to their treatment regimen ^62^. The most common adverse effect related to clofazimine is reversible skin discoloration, which occurs in the majority of patients. Prescribing clofazimine requires the submission of an Investigational New Drug Application to the FDA. Clofazimine should not be used for the treatment of MAC in people living with HIV, as it was associated with increased mortality in this population ^63^.

### Bedaquiline

Bedaquiline is a diarylquinoline that has been recently licensed for the treatment of multidrug-resistant tuberculosis. Bedaquiline has strong *in vitro* activity against MAC ^64^ and lesser activity versus *M. abscessus* ^65, 66^. A study of bedaquiline as salvage therapy in 10 patients with refractory MAC or *M. abscessus* lung disease showed a clinical and microbiologic response in the majority of patients, although none achieved sustained culture conversion ^67^. Subsequent studies have demonstrated the emergence of bedaquiline-resistant isolates ^68, 69^, including seven of 16 bedaquiline-treated patients ^69^. Bedaquiline may cause QT prolongation and other toxicity. Its role in the treatment of refractory NTM infections remains to be defined.

## Future directions

A recent NIH workshop developed a roadmap for research in pulmonary NTM infections ^70^. Among the priority areas that they identified were improved treatment regimens, protocol standardization for whole-genome sequencing analysis, and the development of biomarkers to differentiated initial, persistent, and recurrent infections.

Akin to tuberculosis, the treatment of NTM lung disease requires multiple drugs given for long durations. However, successful treatment outcomes are more difficult to achieve and the risk for disease recurrence is higher in NTM lung disease compared to tuberculosis. Providers should strongly consider the early referral of patients with NTM lung disease to clinicians or centers with expertise in the management of this challenging infection.
